# Soft Tissue Edema Around Musculoskeletal Sarcomas at Magnetic Resonance Imaging

**DOI:** 10.1080/13577149778308

**Published:** 1997-12

**Authors:** David M. Panicek, Lawrence H. Schwartz

**Affiliations:** Department of Radiology Memorial Sloan-Kettering Cancer Center 1275 York Avenue New York NY 10021 UK

## Abstract

The presence of soft tissue edema around a malignant musculoskeletal neoplasm can interfere with accurate local tumor staging at magnetic resonance imaging. This article discusses and illustrates such edema, emphasizing means for avoiding misinterpretation of edema and subsequent overstaging.


*Sarcoma* (1997) 1, 189–191

CARFAX

ISSUES IN IMAGING

### Soft tissue edema around musculoskeletal sarcomas at magnetic resonance imaging

DAVID M. PANICEK & LAWRENCE H. SCHWARTZ

*Department of Radiology, Memorial Sloan-Kettering Cancer Center, New York, USA*

**Abstract**
The presence of soft tissue edema around a malignant musculoskeletal neoplasm can interfere with accurate local tumor staging at magnetic resonance imaging. This article discusses and illustrates such edema, emphasizing means for avoiding misinterpretation of edema and subsequent overstaging.

**Key words:** *sarcoma, staging, soft tissue neoplasms, bone neoplasms, magnetic resonance imaging.*

Accurate definition of the local extent of a sarcoma is required to maximize the success of subsequent interventions while minimizing the amount of tissue (and function) that is removed.[1–5] Due to its excellent ability to distinguish normal from abnormal tissue, magnetic resonance imaging (MRI) is often utilized for local staging of sarcomas. However, an abnormal MRI signal is frequently present in soft tissues surrounding a soft tissue sarcoma or the extraosseous component of a bone sarcoma, characterized by a very high signal on T2-weighted or short tau inversion recovery (STIR) images.[6–8] Such a high signal is due predominantly to an increased water content of the tissues, and is thus generically referred to as 'edema'. (Peritumoral edema is less of a problem during computed tomography (CT) because soft tissue edema is less evident on CT images.) The abnormal MRI signal likely corresponds to the well-known 'reactive zone' present in the tissues around sarcomas,[9] although this has yet to be rigorously proven. Potentially, the reactive zone may contain viable tumor, but the entire reactive zone cannot be excised in every case if the affected limb is to be preserved in a functional state. Peritumoral edema can extend for considerable distances as the fluid is 'squeezed' along by muscle contractions.

As a practical matter, failure to distinguish tumor and peritumoral edema on MRI can result in overstaging of tumor extent (Fig. 1). Such overstaging is particularly problematic with the STIR sequence due to its increased sensitivity to increased water content in a tissue.[10] Therefore, reliance cannot be placed on STIR sequences alone when determining the local extent of a sarcoma.

One helpful MRI feature that has been described is the muscle texture sign.[11] On thin (e.g. 5 mm) axial T1-weighted images of normal muscles, intramuscular fat present between groups of fascicles often produces a typical macroscopic 'marbled' parenchymal pattern, which is usually preserved even in the presence of edema. In contrast, tumours do not exhibit such a pattern of fat within them. Thus, when confronted with an abnormal signal in soft tissues on a T2-weighted image, correlation should be made with the corresponding T1-weighted image obtained at the same anatomic location to determine whether the muscle texture sign can be appreciated in that area. The presence of the muscle texture sign effectively precludes the presence of macroscopic tumor in that area; however, lack of the sign does not prove the presence of tumor.[11]

Also, dynamic intravenous administration of gadolinium-based contrast material may provide differential enhancement of tumor and peritumoral edema.[12] In general, malignant tumours enhance faster than edematous tissue, so that MRI performed during and immediately after rapid intravenous administration of contrast material may cause relatively greater enhancement of the tumor than the peritumoral edema. Of note, non-dynamic imaging is not reliably helpful in this regard.[13,14]

Awareness of peritumoral edema and means to facilitate its recognition is important to avoid overstaging the soft tissue extent of a sarcoma at MRI.

Correspondence to: D. M. Panicek, Department of Radiology, Memorial Sloan-Kettering Cancer Center, 1275 York Avenue, New York, NY 10021, USA. Tel: + 1 212 639 5825; Fax: + 1 212 794 4010; E-mail: panicekd@mskcc.org

1357-714X/97/030189-03 $9.00 © 1997 Carfax Publishing Ltd

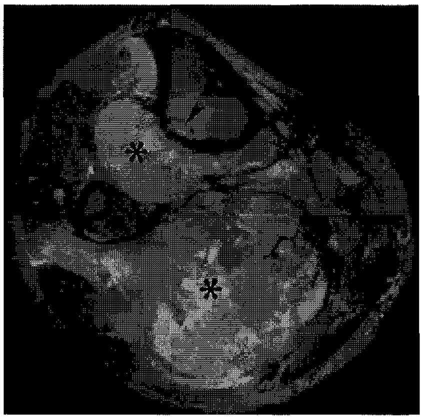

*(a)*

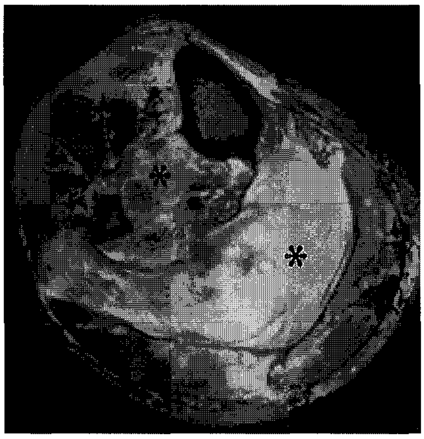

*(b)*

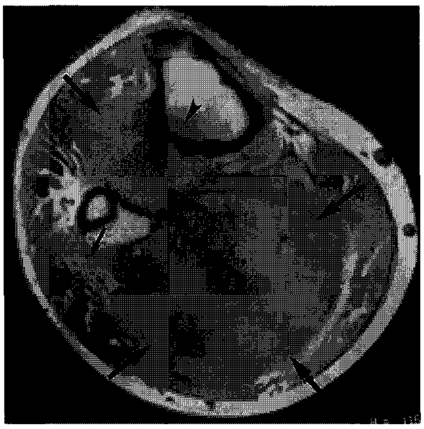

*(c)*

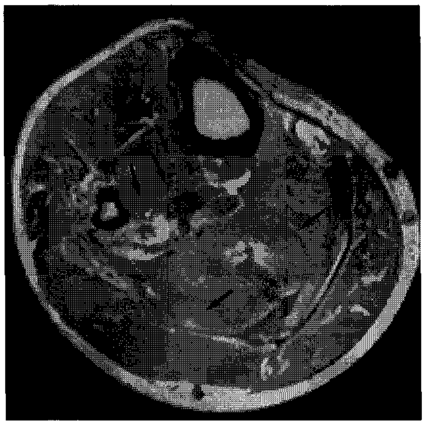

*(d)*

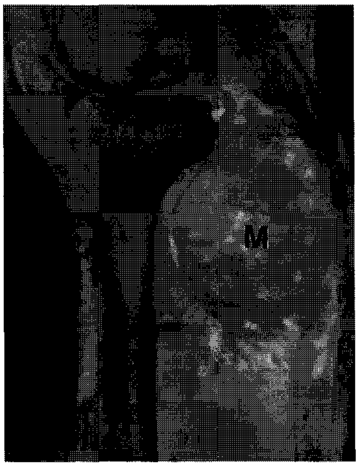

*(e)*

**Fig. 1.** *Clear cell sarcoma (melanoma of soft parts) in proximal calf. (a, b) Axial fat-suppressed fast spin-echo T2-weighted (TR 4000 ms/TE 105 ms) images, with (a) located 3 cm cephalad to (b). Extensive mass-like areas of abnormal high signal (asterisks) are evident in the soft tissues of the proximal calf. Differentiation of tumor and peritumoral edema is not evident on these images alone. (Arrowhead in (a) denotes a tumor deposit within the tibial marrow.) (c) Axial 6-mm thick T1-weighted (700/14) image obtained at same anatomic level as (a) shows large, bilobed tumor mass (long arrows) without evidence of high-signal fat streaked within it. In contrast, note the streaky high-signal fat (short arrows) present within normal muscle near the tumor mass. (Arrowhead in (c) denotes a tumor deposit within the tibial marrow.) (d) Axial 6-mm-thick T1-weighted (700/14) image obtained at same anatomic level as (b) shows normal high-signal fat (arrows) streaked within muscles (i.e. normal muscle texture sign), indicating lack of tumor mass in this region. Abnormal high signal present on the T2-weighted image in (b) thus was due to peritumoral edema. (e) Sagittal T2-weighted (4000/105) image demonstrates the rounded tumor mass (M) with extensive surrounding edema, both having high signal intensity.*

### References

1 Enneking WF, Spanier SS, Goodman MA. The surgical staging of musculoskeletal sarcoma. *J Bone Joint Surg* 1980; 62A:1027–30.

2 Enneking WF, Spanier SS, Malawer MM. The effect of the anatomic setting on the results of surgical procedures for soft parts sarcoma of the thigh. *Cancer* 1981; 47:1005–22.

3 McDonald DJ. Limb-salvage surgery for treatment of sarcomas of the extremities. *AJR* 1994; 163:509–13.

4 Smith DK, Parsons TW. Limb-salvage surgery for treatment of sarcomas of the extremities. *AJR* 1994; 163:514–16.

5 Panicek DM, Go SD, Healey JH, *et al.* Soft-tissue sarcoma involving bone or neurovascular structures: MR imaging prognostic factors. *Radiology* 1997; 205:871–5.

6 Beltran J, Simon DC, Katz W, *et al.* Increased MR signal intensity in skeletal muscle adjacent to malignant tumours: pathologic correlation and clinical relevance. *Radiology* 1987; 162:251–5.

7 Hanna SL, Fletcher BD, Parham DM, *et al.* Muscle edema in musculoskeletal tumours: MR imaging characteristics and clinical significance. *J Magnet Reson Imaging* 1991; 1:441–9.

8 Lang P, Honda G, Roberts T, *et al.* Musculoskeletal neoplasm: perineoplastic edema versus tumor on dynamic postcontrast MR images with spatial mapping of instantaneous enhancement rates. *Radiology* 1995; 197:831–9.

9 Enneking WF, Musculoskeletal Tumor Society. Staging of musculoskeletal neoplasms. *Skeletal Radiol* 1985; 13:183–94.

10 Shuman WP, Patten RM, Baron RL, *et al.* Comparison of STIR and spin-echo MR imaging at 1.5 T in 45 suspected extremity tumours: lesion conspicuity and extent. *Radiology* 1991; 179:247–52.

11 Biondetti PR, Ehman RL. Soft-tissue sarcomas: use of textural patterns in skeletal muscle as a diagnostic feature in postoperative MR imaging. *Radiology* 1992; 183:845–8.

12 Erlemann R, Reiser MF, Peters PE, *et al.* Musculoskeletal neoplasms: static and dynamic Gd-DTPA-enhanced MR imaging. *Radiology* 1989; 171:767–73.

13 Seeger LL, Widoff BE, Bassett LW, *et al.* Preoperative evaluation of osteosarcoma: value of gadopentetate dimeglumine-enhanced MR imaging. *AJR* 1991; 157:347–51.

14 May DA, Good RB, Smith DK, *et al.* MR imaging of musculoskeletal tumours and tumor mimickers with intravenous gadolinium: experience with 242 patients. *Skeletal Radiol* 1997; 26:2–15.
